# The importance of transdiagnostic symptom level assessment to understanding prognosis for depressed adults: analysis of data from six randomised control trials

**DOI:** 10.1186/s12916-021-01971-0

**Published:** 2021-05-06

**Authors:** C. O’Driscoll, J. E. J. Buckman, E. I. Fried, R. Saunders, Z. D. Cohen, G. Ambler, R. J. DeRubeis, S. Gilbody, S. D. Hollon, T. Kendrick, D. Kessler, G. Lewis, E. Watkins, N. Wiles, S. Pilling

**Affiliations:** 1 Centre for Outcomes Research and Effectiveness (CORE), Research Department of Clinical, Educational & Health Psychology, University College London, 1-19 Torrington Place, London, WC1E 7HB UK; 2 iCope – Camden & Islington Psychological Therapies Services, Camden & Islington NHS Foundation Trust, St Pancras Hospital, London, NW1 0PE UK; 3 Department of Clinical Psychology, Leiden University, Leiden, The Netherlands; 4 Department of Psychiatry, University of California, Los Angeles, Los Angeles, CA USA; 5 Statistical Science, University College London, 1-19 Torrington Place, London, WC1E 7HB UK; 6 School of Arts and Sciences, Department of Psychology, 425 S. University Avenue, Philadelphia, PA 19104-60185 USA; 7 Department of Health Sciences, University of York, Seebohm Rowntree Building, Heslington, York, YO10 5DD UK; 8 Department of Psychology, Vanderbilt University, Nashville, TN USA; 9 Primary Care, Population Sciences and Medical Education, Faculty of Medicine, University of Southampton, Aldermoor Health Centre, Southampton, SO16 5ST UK; 10 Centre for Academic Primary Care, Population Health Sciences, Bristol Medical School, University of Bristol, Canynge Hall, Bristol, UK; 11 Division of Psychiatry, University College London, Maple House, London, W1T 7NF UK; 12 Department of Psychology, University of Exeter, Sir Henry Wellcome Building for Mood Disorders Research, Perry Road, Exeter, EX4 4QG UK; 13 Centre for Academic Mental Health, Population Health Sciences, Bristol Medical School, University of Bristol, Oakfield House, Bristol, UK; 14 Camden & Islington NHS Foundation Trust, St Pancras Hospital, 4 St Pancras Way, London, NW1 0PE UK

**Keywords:** Item level analysis, Network modelling, Comorbidity, Depression, Anxiety

## Abstract

**Background:**

Depression is commonly perceived as a single underlying disease with a number of potential treatment options. However, patients with major depression differ dramatically in their symptom presentation and comorbidities, e.g. with anxiety disorders. There are also large variations in treatment outcomes and associations of some anxiety comorbidities with poorer prognoses, but limited understanding as to why, and little information to inform the clinical management of depression. There is a need to improve our understanding of depression, incorporating anxiety comorbidity, and consider the association of a wide range of symptoms with treatment outcomes.

**Method:**

Individual patient data from six RCTs of depressed patients (total *n* = 2858) were used to estimate the differential impact symptoms have on outcomes at three post intervention time points using individual items and sum scores. Symptom networks (graphical Gaussian model) were estimated to explore the functional relations among symptoms of depression and anxiety and compare networks for treatment remitters and those with persistent symptoms to identify potential prognostic indicators.

**Results:**

Item-level prediction performed similarly to sum scores when predicting outcomes at 3 to 4 months and 6 to 8 months, but outperformed sum scores for 9 to 12 months. Pessimism emerged as the most important predictive symptom (relative to all other symptoms), across these time points. In the network structure at study entry, symptoms clustered into physical symptoms, cognitive symptoms, and anxiety symptoms. Sadness, pessimism, and indecision acted as bridges between communities, with sadness and failure/worthlessness being the most central (i.e. interconnected) symptoms. Connectivity of networks at study entry did not differ for future remitters vs. those with persistent symptoms.

**Conclusion:**

The relative importance of specific symptoms in association with outcomes and the interactions within the network highlight the value of transdiagnostic assessment and formulation of symptoms to both treatment and prognosis. We discuss the potential for complementary statistical approaches to improve our understanding of psychopathology.

**Supplementary Information:**

The online version contains supplementary material available at 10.1186/s12916-021-01971-0.

## Background

Psychological therapies and medication are effective treatments for depression (e.g. [1, 2]). However, effect sizes have been modest and gains in treatment outcomes have plateaued [3]. Interventions for depression target a broad range of symptoms, and knowledge of ‘what’ is being intervened upon is not necessary to the delivery of most treatments, and poses problems for causal inference [4]. To improve interventions, we may need to improve our knowledge of the structure of depression [5].

Depression is heterogeneous in terms of aetiology and symptom profile [6–8]. Mood disorders are highly comorbid with anxiety disorders and may share psychological and biological vulnerabilities [9, 10]. The risk of one disorder can increase the risk of another [11], and the same end state may be achieved via many different paths (equifinality) [12, 13]. These disorders are not discrete entities and, as such, neglecting the symptomatic heterogeneity discards potential insights [14].

There is strong evidence that different symptoms are not equivalent or interchangeable [15] and studies of individual symptoms in the last decade have brought important understanding. For example, individual symptoms may differ in response to treatment [16, 17] and have been shown to have a differential impact on functioning [18–20]. Depression is a recurrent disorder with the probability of relapse strongly associated with the presence of residual depressive symptoms at the end of treatment [21, 22]. Comorbid anxiety disorders are related both to worse treatment outcomes [23] and to an increased risk of relapse [21]. An assumed unidimensional view of depression, characterised by sum score (sum of symptom severity scores) measurement and prediction models, conceals the variability within depression [24]. Understanding the relative importance of comorbid symptoms may offer more information than severity of disorder alone and provide additional treatment and prognostic information [25]. Large-scale, multisite clinical trial data, coupled with innovative statistical methods, can provide categorisation and treatment optimisation to provide immediate benefits by informing clinical decisions [26–28].

There is also value in studying the relations among these symptoms. Network theory posits that the relationships between common affective, cognitive, and somatic symptoms of these disorders may reflect potential causal pathways and elucidate maintenance mechanisms [29]. Depression and anxiety have been modelled as symptom networks using cross-sectional and longitudinal data, demonstrating the interrelation between the symptoms of each disorder, where comorbidity results from mutually reinforcing interrelation between symptoms of each disorder [30, 31]. Anhedonia, anxiety, worry, fatigue, and sadness are predominantly influential symptoms in these networks [5, 32, 33]. The relationship between symptoms/mechanisms can help to predict outcome and potentially inform treatment targets and the development of treatments targeting specific mechanisms [34].

There are inconsistencies in the network literature exploring depression and anxiety, due to design, sampling, and variability arising from differing measurement [15, 35]. When attempting to discriminate between groups for the purposes of identifying whom may benefit from treatment (prognosis at group level), there are varying results from network comparison studies, where it has been suggested that densely connected networks may be less likely to recover [36]. However, these differences are not always observed [37] and require large sample sizes to detect any effect. It is also unclear how these networks generalise to idiographic networks at the present stage. Past research has been conducted on small samples with low quality assessment of patients (or non-clinical samples) and lack of adequate consideration of comorbidity (missing out on the wider spectrum of anxiety disorders).

In this study we aim to:

1. Identify important symptoms for outcome by examining the (differential) impact of individual symptoms on prognosis for adults with depression that took part in randomised controlled trials after seeking treatment in primary care and assess whether individual symptoms offer predictive value above sum scores.
2. Discern the functional relations among symptoms and clarify the interplay between highly comorbid symptoms of depression and anxiety disorders.
3. Consider whether there are differences in the baseline symptom networks of patients that remitted vs. those whose depression persisted, after treatment.

## Method

### Datasets

Data were drawn from a subset of the Dep-GP individual patient data (IPD) database [36]. The formation of the Dep-GP IPD dataset has been described elsewhere [36]. Bibliographic databases were searched up to 29 April 2020 for RCTs of unipolar depressed adults seeking treatment for depression or with depressive symptoms significant enough for them to seek treatment, recruited from primary care; had at least one active treatment arm; and used the CIS-R at baseline.

Studies were excluded if they were studies of patients with depression secondary to a diagnosis of personality disorder, psychotic conditions, or neurological conditions; bi-polar or psychotic depressions; children or adolescents; feasibility studies; or were studies of adults with either depression or an anxiety disorder, rather than a primary depression with or without comorbid anxiety. Additional inclusion criteria for the present study were the use of the Beck Depression Inventory (2nd Edition) (BDI-II) [37] at study entry. The inclusion criteria ensured uniformity in the measurement of depressive and anxiety symptoms, chronicity of problems, and determination of diagnoses including anxiety comorbidities.

Data on all individual patients from all six eligible RCTs were included in the current study, these were COBALT [38], GENPOD [39], PANDA [40], TREAD [41], MIR [42], and IPCRESS [43].

### Measures

Individual items from the BDI-II [37], and individual symptom subscales of the CIS-R [44], including duration of depression and anxiety, which have been shown to be independently associated with prognosis for depressed adults [45].

### Outcomes

The primary outcome was endpoint depressive symptoms at three to four months post-study entry. Five of the studies used the BDI-II at 3–4 months, and one used the PHQ-9. A continuous ‘depression severity’ score was developed by converting the responses on each measure to a latent trait depressive symptom severity score (PROMIS T-Score) [46], using the expected a posteriori parameter from a multidimensional item-response theory based score conversion tool [47]. Depressive symptoms (PROMIS T-Score) at 6–8 months post-study entry, and 9–12 months were secondary outcomes.

As a sensitivity analysis, the BDI-II scores were used as outcomes for the three time points (five studies at 3–4months; four studies at 6–8months, and three studies at 9–12 months).

### Data analysis

All analyses were performed in R 3.6 [48] and Stata 16.0 [49]. Analysis code is available from https://osf.io/wck6b/. The data that support the findings of this study are available from the lead author of the Dep-GP (JB) subject to agreement from the chief investigators or data controllers of the individual RCTs. Restrictions apply to the availability of these data, which were used under licence for this study.

### Pre-processing

Datasets were combined and pre-processed together. There was no missing data at study entry. All items were investigated to ensure they met assumptions for inclusion in the network models, including assessing for near zero variance, roughly equal variance of items, asymmetrical distributions, and topological overlap [50]. Items were removed if they violated assumptions across all studies. We aimed to address topological overlap using the ‘goldbricker’ function in R [51] with a threshold of 25% (correlations between items should have significantly different correlations with 25% of the other items), accepting minimal correlation of 0.5.The respective pair of items were combined into a single variable using principal component analysis (PCA) if reasonable to combine from a clinical perspective. Items were afterwards rescaled to their original Likert scale values to make variances comparable across items [52].

### Association with outcomes

We aimed to examine the differential impact of individual symptoms on outcomes and assess whether individual symptoms offer predictive value above sum scores. Sum score totals were entered into a linear regression model, while the item severity scores were entered into an elastic net generalised linear model (ENR) [53]. ENR, a statistical method combining lasso and ridge regression approaches, minimises overfitting and the use of ten separate, tenfold repeated cross validation aids in assessing the effectiveness of the model. The item-level and sum-score models were compared using root mean squared error, mean absolute error, and *R*^2^.

As the item-level predictors were assumed to be correlated and that we wished to assess the explanatory power of individual predictors, we estimated the contribution of each item to the outcome prediction using Shapley Additive exPlanations [54], following ENR model estimation. Five hundred Monte Carlo repetitions were used to estimate each Shapley value. This metric is more accurate than other variable importance metrics when predictors are dependent [55]. Items with large Shapley values are ‘important’, indicating the relative contribution of an item to the model while accounting for correlated features in the data.

### Network modelling

A graphical Gaussian model (GGM) aims to capture the direct effects (edges) between items while controlling for all other items in the network. A network was estimated by combining data from the six RCTs. The sample was then split into two networks (those with persistent symptoms vs. remitters: BDI-II score < 10 at 3–4 months); the networks were re-estimated and compared using the network comparison test with 1000 iterations [56].

We performed a number of analyses to test the robustness of the networks we estimated.

While lasso [57], regularised GGMs [58] are most frequently reported in the network literature, lasso specificity has recently been shown to be lower than expected in dense networks with many small edges, leading to an increase in false positives [59]. We also estimated an unregularized GGM using an iterative modelling procedure: the Extended Bayesian Information Criterion (EBIC). Selecting unregularized GGMs according to EBIC has been shown to converge to the true model [60]. The algorithm runs 100 glasso models, re-fits all models without regularisation,, and subsequently adds and removes edges until EBIC can no longer be improved. The best performing model (EBIC parameter) was selected to provide a conservative GGM estimation (high specificity).

Chronicity of disorders has been shown to interact with symptom severity [45, 61]. We corrected for the potential confounding effects of duration of depression and anxiety within the network models.

Combining data obtained from different studies holds the potential for between-study differences to influence estimation. A network estimation procedure (fused graphical lasso: FGL) [62] has been designed to manage this issue, however, this involves estimating networks individually and penalising between study differences. Where study size affects the estimation of edges, this can lead to penalization based on sample size rather than on true differences between the network structures [63]. As such, it was decided to estimate based on the combined sample and to compare this to the FGL network (joint estimation using a fused penalty, and 10-fold cross validation), to assess the potential influence of group level differences.

Finally, the network model was tested for the stability of expected influence centrality and the accuracy of interrelations using a nonparametric bootstrapping procedure (1000 iterations) [64]. For details of these, see the Supplementary material.

We obtained two types of information from the resulting network structures. First, symptoms can form clusters or communities with other symptoms to which they are connected reflecting commonalities between them. We estimated the community structure by using a bootstrapped walktrap algorithm [65], investigated for item stability before selecting communities. Second, the overall connectivity of a symptom, i.e. its connection to other symptoms, can be quantified in a number of ways and is referred to as centrality. Some scholars have argued that activation of a central symptom has the potential to activate associated symptoms in the network [66], where symptom centrality is then interpreted as symptom importance, given that identifying such symptoms may have the potential to elucidate the processes underlying comorbidity and implications for treatment. Within the context of communities specifically, symptoms which connected to more than one community are referred to as bridge symptoms. Within cross-sectional networks (as explored here), we refer to centrality as a statistical parameter, i.e. the strength of predictive associations between symptoms. Centrality does not automatically translate into clinical relevance [67] and cautious interpretation is warranted [63]. It requires consideration of how the symptoms activate within the network (flow or transfer), the conceptual similarity between symptoms, and whether there is missing information on the shared variance [68]. Symptom centrality was calculated using expected influence (EI: strength of the relationships a given node has with other node) and the geometric mean of the participation ratio (PR) and participation coefficient (PC), and normalised bridge expected influence centrality [69]. The PR quantifies the number and strength of edges, while the PC takes the community structure into account [70].

## Results

Demographic details for the studies are presented in Table 1. Overall samples were comparable. The severity of depressive symptoms captured by BDI-II scores at baseline in the PANDA sample was lower than the other trials. Descriptive results are reported in the supplementary materials.

**Table 1:** Descriptive table of studies included in the dataset. Summary of included variables provided in supplementary materials. * International Baccalaureate equivalent ** High school diploma equivalent

	COBALT	GENPOD	IPCRESS	MIR	PANDA	TREAD	Overall
	(***N*** = 469)	(***N*** = 601)	(***N*** = 295)	(***N*** = 480)	(***N*** = 652)	(***N*** = 361)	(***N*** = 2858)
**Baseline BDI-II total**							
Mean (SD)	31.8 (10.7)	33.7 (9.67)	33.2 (8.80)	31.1 (9.91)	23.9 (10.3)	32.1 (9.24)	30.4 (10.5)
Median [min, max]	30.0 [14.0, 60.0]	33.0 [15.0, 60.0]	33.0 [15.0, 58.0]	30.0 [14.0, 58.0]	23.0 [2.00, 54.0]	31.0 [14.0, 57.0]	30.0 [2.00, 60.0]
**Gender**							
Female	339 (72.3%)	408 (67.9%)	200 (67.8%)	332 (69.2%)	384 (58.9%)	239 (66.2%)	1902 (66.6%)
Male	130 (27.7%)	193 (32.1%)	95 (32.2%)	148 (30.8%)	268 (41.1%)	122 (33.8%)	956 (33.4%)
**Age**							
Mean (SD)	49.6 (11.7)	38.8 (12.4)	34.9 (11.6)	50.7 (13.2)	39.7 (15.0)	39.8 (12.6)	42.5 (14.1)
Median [min, max]	50.0 [18.0, 74.0]	38.0 [18.0, 74.0]	34.0 [18.8, 74.6]	51.0 [19.0, 84.0]	38.5 [18.0, 73.0]	39.0 [18.0, 69.0]	42.0 [18.0, 84.0]
**Employment status**							
Employed	206 (43.9%)	357 (59.4%)	178 (60.3%)	237 (49.4%)	433 (66.4%)	230 (63.7%)	1641 (57.4%)
Seeking employment	151 (32.2%)	123 (20.5%)	35 (11.9%)	102 (21.2%)	73 (11.2%)	48 (13.3%)	532 (18.6%)
Not seeking employment	112 (23.9%)	121 (20.1%)	82 (27.8%)	141 (29.4%)	146 (22.4%)	83 (23.0%)	685 (24.0%)
**Education**							
Degree or higher	95 (20.3%)	0 (0%)	102 (34.6%)	95 (19.8%)	230 (35.3%)	87 (24.1%)	609 (21.3%)
A-level or diplomas*	123 (26.2%)	0 (0%)	88 (29.8%)	135 (28.1%)	220 (33.7%)	104 (28.8%)	670 (23.4%)
GCSE**	131 (27.9%)	0 (0%)	62 (21.0%)	150 (31.2%)	145 (22.2%)	102 (28.3%)	590 (20.6%)
None or other	120 (25.6%)	0 (0%)	43 (14.6%)	100 (20.8%)	57 (8.7%)	68 (18.8%)	388 (13.6%)
Missing	0 (0%)	601 (100%)	0 (0%)	0 (0%)	0 (0%)	0 (0%)	601 (21.0%)
**Ethnicity**							
White	459 (97.9%)	575 (95.7%)	281 (95.3%)	469 (97.7%)	579 (88.8%)	336 (93.1%)	2699 (94.4%)
Non-White	10 (2.1%)	26 (4.3%)	14 (4.7%)	11 (2.3%)	73 (11.2%)	25 (6.9%)	159 (5.6%)
**Diagnoses**							
Number of comorbid diagnoses	2.40 (1.09)	2.39 (0.92)	2.32 (0.99)	2.10 (0.97)	1.43 (1.18)	2.20 (1.17)	2.09 (1.12)
Generalised anxiety disorder	312 (66.52%)	410 (68.22%)	186 (63.05%)	219 (45.63%)	299 (45.86%)	238 (65.93%)	1664 (58.2%)
OCD	79 (16.84%)	114 (18.97%)	62 (21.02%)	62 (12.92%)	52 (7.98%)	50 (13.85%)	419 (14.7%)
Panic disorder	67 (14.29%)	51 (8.49%)	16 (5.42%)	45 (9.38%)	42 (6.44%)	14 (3.88%)	235 (8.2%)
Agoraphobia	61 (13.01%)	75 (12.48%)	28 (9.49%)	81 (16.88%)	42 (6.44%)	35 (9.70%)	322 (11.3%)
Social phobia	64 (13.65%)	64 (10.65%)	44 (14.92%)	58 (12.08%)	68 (10.43%)	52 (14.40%)	350 (12.2%)
Specific phobias	91 (19.40%)	127 (21.13%)	46 (15.59%)	62 (12.92%)	98 (15.03%)	61 (16.90%)	485 (17%)
Chronic fatigue syndrome	343 (73.13%)	476 (79.20%)	220 (74.58%)	311 (64.79%)	288 (44.17%)	257 (71.19%)	1895 (66.3%)

### Association with outcomes

In order to assess the utility of item level models, we compared them to sum score models. For all item level models (Table 2), the optimal shrinkage parameters for the elastic net regression model were selected via minimum cross-validated error criterion (*ᾳ* = 0.1 and *λ* = 0.05). While models performed similarly at 3–4 months and 6–8 months, the item level elastic net regression model outperformed linear regression with BDI-II and CIS-R (sum of anxiety items) totals at the 9–12-month time point. The sensitivity analysis performed similarly. Due to the absence of two studies (IPCRESS and PANDA) at the 9–12-month endpoint, we reran the analyses for the earlier time points without these studies. This sensitivity analysis did not reveal any difference in the pattern of model performance.

**Table 2:** Performance of the regression models. Sum scores: BDI-II and CIS-R; RMSE root mean squared error; MAE mean absolute error; *R*^2^ proportion of the variance explained

		PROMIS T-score		
		RMSE	*R*^2^	MAE
3 to 4 months *N* = 2646	Items	0.925	0.146	0.73
	Sum scores	0.926	0.143	0.73
6 to 8 months *N* = 1297	Items	0.926	0.147	0.734
	Sum scores	0.924	0.146	0.735
9 to 12 months *N* = 1110	Items	0.919	0.161	0.744
	Sum scores	0.935	0.126	0.753

**Fig. 1:**
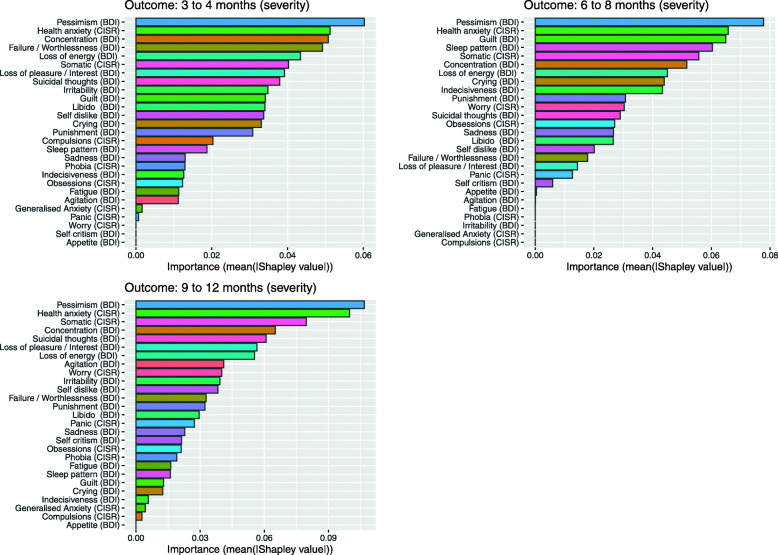
Shapley values for variable importance are plotted: (showing the difference contribution of items to predictions)

Pessimism (Fig. 1) was consistently the most important item; health anxiety was in the upper quartile at each time point; and concentration, failure/worthlessness, also in the upper quartile at 3–4months; guilt and sleep at 6–8 months; and somatic symptoms at 9–12 months.

### Network modelling

For the individual items in the network model, near zero variance (e.g. due to floor and ceiling effects) was not observed. However, we saw asymmetric distributions (skew) on a number of items. As such, a Spearman covariance matrix was estimated and used to estimate the network model. Multi-collinearity was identified for two pairs of items (loss of pleasure with loss of interest, failure with worthlessness). New items were constructed using PCA for each pair. The optimal model for the network analysis was an unregularized graphical Gaussian model using the EBIC.

A walktrap algorithm identified three, stable, symptom communities (median = 3, SD = 0.15, 95% CI [2.71, 3.29]). The three communities split into anxiety items, depressive cognitions and depressive physical symptoms. Bridging EI elucidated three bridging symptoms between the communities: sadness and indecisiveness (from the physical symptoms community) and pessimism (cognitive symptoms community).

**Fig. 2:**
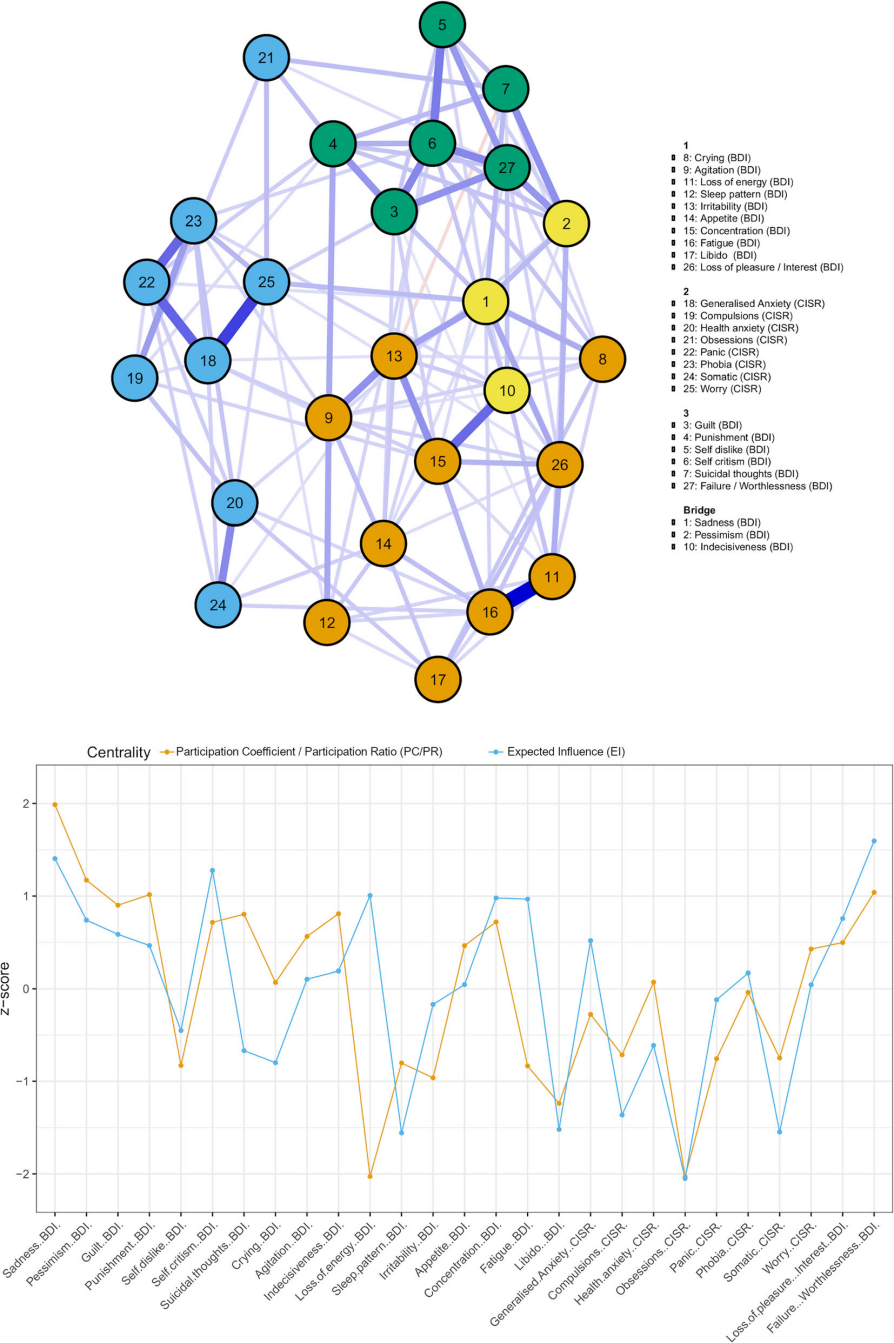
Network plot (top) with communities. Bridge symptoms are categorised separately; however, sadness and indecisiveness fall into community 1, and pessimism into community 3. The thickness of the edges indicates to what degree items are related, and the colour of the edges indicates the relationship sign (i.e. positive = blue, negative = red). Centrality estimates: PC/PR and EI (bottom)

Centrality estimates (i.e. measures of the strength of connection to other items) are reported in Fig. 2. The EI correlation stability coefficient was high (0.75), suggesting that the ordering of items based on centrality remained the same after re-estimating the network with fewer cases (the probability the correlation between original centrality indices and centrality of networks based on subsets was 0.7 or higher) and can be reliably interpreted.

The estimates from the different metrics (EI and PC/PR) were correlated (*r* = 0.58). The most central symptoms were sadness (PC/PR) and failure/worthlessness (EI). Failure/worthlessness had a significantly higher EI centrality than twenty-one other symptoms (see supplementary material). The next most central nodes (EI) were sadness, self-criticism, and loss of energy (all *z*-score > 1), followed by concentration, loss of pleasure/interest, and fatigue (*z*-score > 0.96), while the next most central nodes when using PC/PR were pessimism, failure/worthlessness, and punishment (all *z*-score > 1), then guilt, indecisiveness, and suicidal thoughts (all *z*-score > 0.80). Notably, while suicidal thoughts were highly central according the PC/PR metric (*z*-score = 0.80), it was much less central using EI (*z*-score = − 0.67). Loss of energy displayed the opposite relationship, more central for EI (*z*-score = 1.01) than PC/PR (*z*-score = − 2.03). Loss of energy and obsessions were jointly the least central nodes using PC/PR, and obsessions was also the least central when using EI.

Robustness checks suggest the resulting graphical Gaussian model was stable and accurate. Stability and accuracy plots, individual networks (with the fused penalty) and the fused network model are supplied in the supplementary materials. Mean severity was not significantly correlated (*p* < 0.05) with EI (*r* = 0.21) or PC/PR (*r* = − 0.05), while the standard deviation was significantly correlated for both EI (*r* = − 0.56) and PC/PR (*r* = − 0.41). Symptom severity was not associated with nodes being interconnected. Lower variability was associated with variability, which is the reverse of a more typical concern: differential variability driving the centrality of nodes [52].

The interrelation of the network and the FGL network were compared (*r* = 0.72), suggesting that between study differences had a small effect on network estimation. The network was corrected for the influence of duration of depression and anxiety; however, the overall influence on edge estimation was negligible (interrelation between the corrected network and a network estimated without duration variables: *r* = 0.997). Overall, the resulting network model can be considered robust.

### Network comparison test

Networks (unregularized) were compared (1000 iterations) for those who were classified as in remission (*n* = 956) and those who were not (*n* = 1466). Mean severity differences at baseline were significant for all items (*p* < 0.001). The correlation between networks was high (*r* = 0.67). While there were significant difference between edges, the overall networks (see supplementary material) did not differ in connectivity (global strength invariance: *p* < 0.08) and post hoc tests were not warranted. There was only evidence of one difference in centrality between the networks: somatic symptoms were more connected in the remitter network than the persister network (*p* < 0.001).

## Discussion

Individuals with depression also present with comorbidity, and this could present an issue for depression treatment. Understanding how symptoms influence one another across traditional diagnostic boundaries, and how they influence important outcomes, may provide insights relevant to the assessment and treatment of mood disorders. In this study, we initially examined the differential impact of individual symptoms on prognosis and assessed whether individual symptoms offer predictive value above sum scores. The item level models of outcomes post-treatment and the sum score models were similarly associated with outcomes at 3–4 and 6–8 months but explained considerably more variance at 9–12 months. Pessimism was consistently the most important predictor of future outcomes (independent of its mean), indicating that experiencing pessimism rather than severity of the symptom is responsible for this association. Secondly, we explored the functional relations among comorbid symptoms of depression and anxiety disorders using network analysis. The symptom network comprised of three communities clearly clustering into anxiety items, depressive cognitions, and depressive physical symptoms. The primary bridge symptoms between communities were sadness; pessimism; and indecision. The most central symptoms across both centrality metrics were sadness and failure/worthless. Finally, we analysed differences in the symptom networks at study entry for patients that remitted vs. those whose depression persisted, after treatment. Network comparison revealed no overall differences in connectivity. Together, the present findings suggest the utility of item-level analysis in informing the content of assessments and consideration of individual items over and above scale scores when predicting prognosis.

### Findings in context

Exploring the associations with treatment outcomes revealed that item-level prediction methods performed similarly to sum scores and outperformed sum score models at the 9–12-month endpoint. It is not clear why there is a difference at this time point; while it was not due to attrition between endpoints, it could be due to random variation. It may also reflect the course of depression following intervention, or the cyclical nature of depression such that individual items are better at predicting the relapse or maintenance of symptoms after benefits of treatment have faded, or where an amelioration of symptoms occurred due to further treatment post randomisation. There is an ongoing debate in the field whether central items derived from network models offer predictive utility beyond other items [71–73]. Pessimism was not only the best predictor across outcomes; it was a central item (ranked 2nd on PC/PR and 6th on EI centrality) that acted as a bridge between communities and showed strong associations with sadness and failure/worthlessness. Sadness, comparatively, did not predict well across time points. It is worth noting that sadness falls within the physical symptom community and pessimism within the cognitive community. The amenability to act on an emotion (sadness) is understandably less than that of a cognition (pessimism), a target of cognitive therapy, while pessimism in association with a sense of failure/worthlessness may negatively impact treatment engagement (i.e. the motivation to sustain goal pursuit in the face of obstacles) [74]. Given the central role and prognostic value of pessimism, we might speculate that it is associated with treatment factors, where pessimism hinders some people making progress and may not be directly addressed by some psychological treatments.

Symptoms of anxiety and depression clustered into separate communities with certain symptoms acting as bridges between communities. The bridge symptoms are statistically relevant and theoretically linked: indecision is a symptom in the classifications of both depression and generalised anxiety disorder, pessimism overlaps with worry [75] and the strong cross-community edge of sadness to worry was similar to findings in other studies [32, 76]. The results therefore provide evidence that these bridging symptoms may be important in the emergence of comorbidity between anxiety disorders and depression.

Planned comparisons of networks at study entry for those whose depression would persist versus those who would be in remission revealed no overall difference in connectivity, in contrast to Van Borkulo et al. [77], but similar to Schweren et al. [78].

Overall, we found no correlation between centrality metrics and Shapley values. This extends prior work on the association between centrality and the prognostic utility of items [71]. Failure/worthlessness was predictively important at 3–4 months, displayed high centrality and is suggested to be a key symptom in depression and anxiety [30]. The predictive utility of health anxiety and somatic concerns may be considered alongside the observation from the network comparison where there was a difference in centrality with somatic concerns more connected in the remitter network. Health anxiety was in the upper quintile of variable importance across time points, but relatively unimportant in terms of centrality. Not surprisingly, given the conceptual overlap, with health anxiety, the strongest edge was with somatic concerns. As such, the degree of concern for one’s health, or attention to somatic symptoms, whilst not playing a significant role within the maintenance of depression, may act as a motivational spur to engage with treatment (in this way enabling rather than disabling the individual). The absence of this anxiety may reflect an apathy about one’s health which is not captured by the motivational item in the BDI. While the predictive modelling did consider the influence of each item independent of the other items, modelling the predictive value of individual items may be improved by examining the association between the changes at symptom level and the overall network [79, 80].

The network derived in this study provides empirical phenomena that can be explained by principles of network theory. This requires interpreting the network as a causal system, even though we cannot infer temporal relationship between symptoms and there is an absence of causal mechanisms within the external field (e.g. environmental factors) [29]. These limitations apply to most of the findings in the network literature, although overinterpretation is common [81]. Holding this in mind, we can consider possible pathways and mediating role of symptoms through the network. For example, taking suicidal ideation as a clinically severe symptom, we can identify the shortest path from worry [82] passing through sadness (bridge), and from loss of pleasure/interest [83] to suicidal thoughts, passed through pessimism (bridge). It is possible that any causal effect between these connections may be part of a longer pathway within the network highlighting a need for greater attention to be given to symptom interactions.

The statistical model investigates a symptom level, transdiagnostic conceptualization of the symptom interactions for individuals diagnosed with depression participating in RCTs. These interventions are based on biological or psychological theories, most notably Beck’s cognitive of theory of depression [84]. Clinically, pragmatism trumps theoretical completeness; simple interventions which achieve rapid change do not require a detailed appreciation of the potential underlying mechanisms. However, oversimplified theories may restrict the ability to identify causal patterns, and gaps emerge in practice where the model is suggested to not fit the patient [85]. More process-driven interventions targeting shared features of disorders have been developed [86, 87], yet there is no unifying theory. The findings presented may help bridge the gap between disorder-specific theories and more transdiagnostic theories. Considering how symptoms may interact can help clinicians and researchers to understand underlying processes and in turn to conceptualise their patients’ difficulties in a way that supplements existing knowledge. A functional analysis which integrates the association between sadness and worry does not need to conceptualise the individual as having two disorders, but can consider how, for the individual, this interaction is being fuelled and may be contributing to their distress.

The journey to develop models that provide both explanatory and predictive utility will lead to greater understanding of psychopathology [88]. While the analysis presented is primarily exploratory, it sets up clear testable hypotheses. These can be derived by examining the central structures within the network, formulating hypotheses and testing on an independent sample [89]. For instance, whether the bridge edges belonging to pessimism, sadness, and indecisiveness re-emerge in an independent sample or whether a discrete intervention targeting pessimism would alter the network structure and lead to improved outcome. These statistical methods may help inform how identifying pathways and targets may lead to improved treatments all dependent on better assessment of symptoms.

### Strengths and limitations

This study has clear strengths, making use of a large sample of individuals participating in RCTs for depression in primary care. The use of same assessment measures at study entry removed the need to harmonise data across different measures for the network. While this is less true of outcomes where issues of measurement errors arise from the use of PROMIS T-Score, the sensitivity analyses provided confidence in the results.

The demographic balance across samples may affect generalisability; however, five of the six trials were pragmatic trials more closely representative of patient populations. Most cases of depression are treated in primary care, and the studies being set in primary care, improve the potential generalisability to patients seen in this setting [90].

This study was limited to the use of aggregate/group level findings to inform within person processes. However, the presence of an RCT outcome variable affords us the ability to detect changes from one state (e.g. depressed) to another (e.g. remitted), which is typically not the case with idiographic research studies that collect cross-sectional data. Exploring the prognostics value of networks on deterioration of symptoms would extend the utility of network analysis. This would however require generating idiographic networks, where reliable estimation necessitates many time points (low sensitivity at 100 time points [91].

The accuracy of the network is limited by the items included and those omitted. The network does not cover the breadth of comorbidity of symptoms across psychopathology and is missing other environmental variables. Social adversity is associated with worse treatment outcomes for some patients with depression; it can be important to assess for and address these issues in clinic, where possible, to mitigate the risks of poor prognoses [92]. There is also the possibility that the centrality of sadness particularly represents a strong association with a latent variable rather than a specific role within the network [93].

The network models adjusted for duration of depression and anxiety, and a sensitivity analysis assessed for the influence of between study variability, adding robustness to the findings. While RCTs are used in the analysis, treatment arms were not factored in and treated as equivalent when estimating outcome. This may make the findings generalizable where findings are applicable regardless of treatment offered especially as the treatments included reflect those commonly available in primary care. Controlling for treatment group within the outcome modelling and controlling for relevant covariates (e.g. age, gender and social economic status) would also have improved the robustness of the findings. Such adjustments would have been fitting where the emphasis was on developing the best predictive model, instead of comparing the predictive ability of symptoms vs. total scores. More comprehensive prediction modelling using the Dep-GP dataset has been conducted [94]. Additionally, our modelling did not include train/test split, as the whole sample was used in estimation of the network models. While a true out-of-sample ‘holdout’ dataset would have provided an unbiased evaluation of model fit, and is the preferred method for evaluating such models [95], the internal cross-fold validation employed in the symptom level model offers a layer of robustness supporting the final model estimates (where overfitting presents an issue). This study focussed on item-level analysis in comparison to sum-scores, future comparisons with models which may measure latent constructs in other ways, could be informative.

Single item symptom measurement will have unknown reliability and construct validity. Equally, the restricted range (e.g. a four-point scale) may not adequately capture the range of symptom variance occurring in the sample. Symptom measurement on a broader scale may improve the prediction of changes over time.

## Conclusions

Our study used samples from high-quality randomised controlled trials, and the findings can be generalised to adults with depression being treated in primary care. This study has reiterated the importance of assessing for both depressive and anxious symptoms among adults seeking treatment for depression, and that valuable information about prognosis can be gained by understanding the interrelations between individual symptoms, information which is not available when considering sum scores or baseline symptom severity alone. This may be particularly important to longer term outcomes from treatment. Treatment selection and application is often hampered by comorbid symptoms and considered to introduce ‘complexity’ [96]. Considering the bidirectional relationship between symptoms and associations which may be mediated by another symptom (e.g. a bridge symptom) may help to consider comorbidity as normative.

While specific symptoms and associations have been highlighted, the aim is not to offer simple heuristics to inform clinical judgement and decision making. The relative importance of the highlighted associations should not be overweighed. The aim is not to identify individual items, but to consider the network of interactions. The critical role of individual symptoms and their interactions give rise to the activation of the network through pathways and anxiety and depressive cognitive and physical symptoms may activate one another via these pathways. This network highlights how symptoms of depression and anxiety disorders influence one another. Clinically, there is a need for treatments to adequately assess and address comorbidity.

## Supplementary Information

**Additional file 1.**

## Data Availability

The data that support the findings of this study are available from the authors of the individual trial studies. Restrictions apply to the availability of these data, which were used under licence for the current study, and so are not publicly available.

## Abbreviations

BDI-II: Beck depression invention (2nd edition)
CIS-R: Revised Clinical Interview Schedule
EBIC: Extended Bayesian information criterion
EI: Expected influence
ENR: Elastic net regression
FGL: Fused graphical lasso
GGM: Gaussian graphical model
IPD: Individual patient data
MAE: Mean absolute error
PC: Participation coefficient
PCA: Principal component analysis
PHQ-9: Patient Health Questionnaire 9
PR: Participation ratio
RMSE: Root mean squared error
