# Supplementary material for: The importance of transdiagnostic symptom level assessment to understanding prognosis for depressed adults: analysis of data from six randomised control trials
Source: BMC Med. 2021 May 6;19:109. doi: 10.1186/s12916-021-01971-0 (PMC8101158; doi:10.1186/s12916-021-01971-0)
Supplement: Supplementary file 1 — Additional file 1. [file 12916_2021_1971_MOESM1_ESM.docx]

Supplementary material


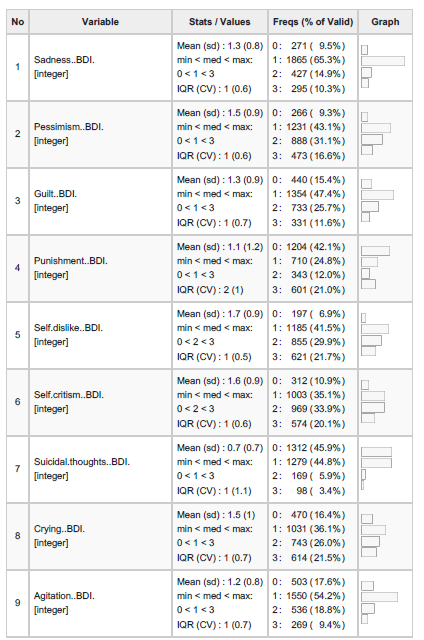

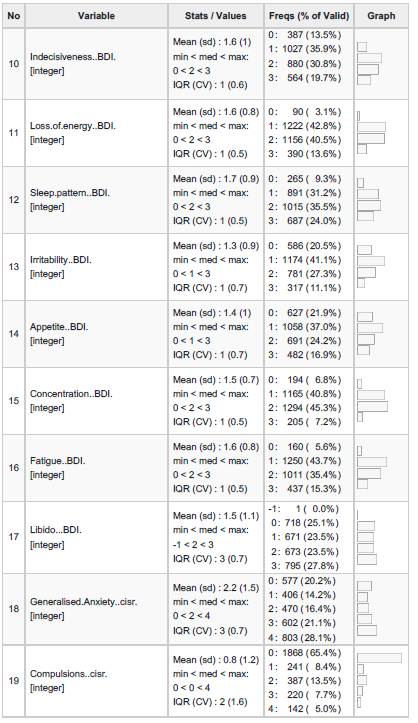

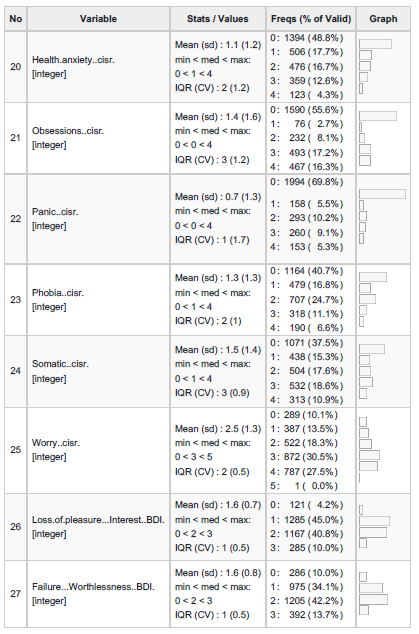
Item level descriptive data.


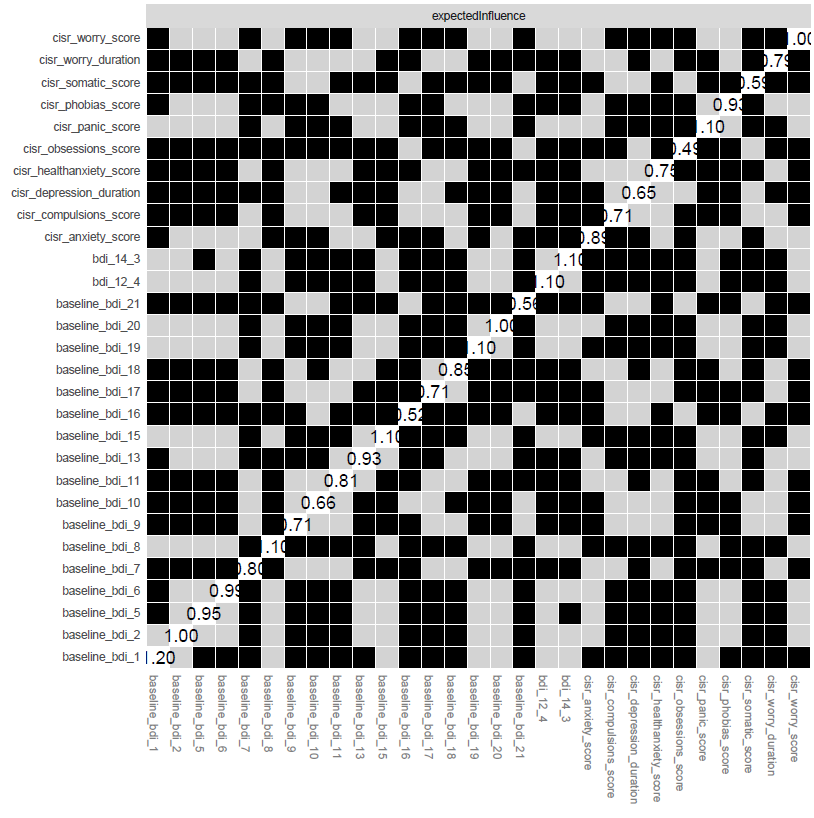
Expected influence: significant difference.


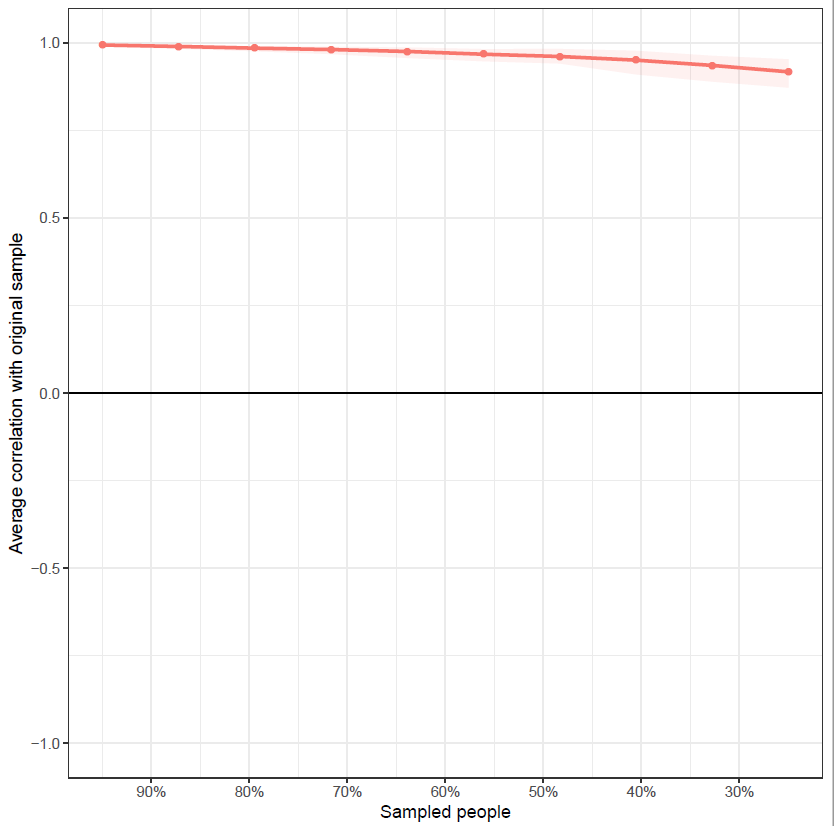


Expected influence stability.


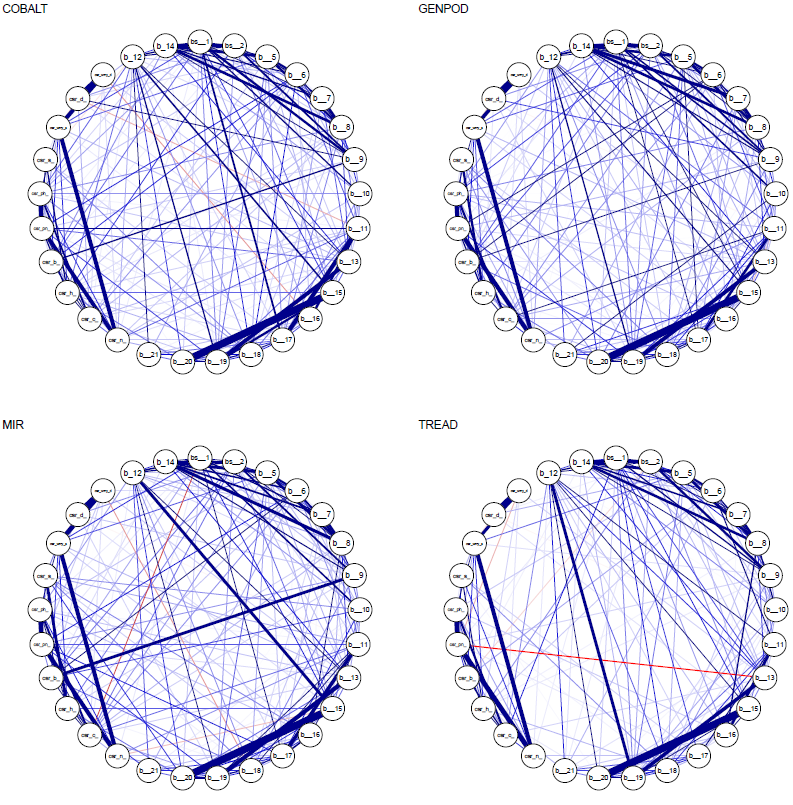

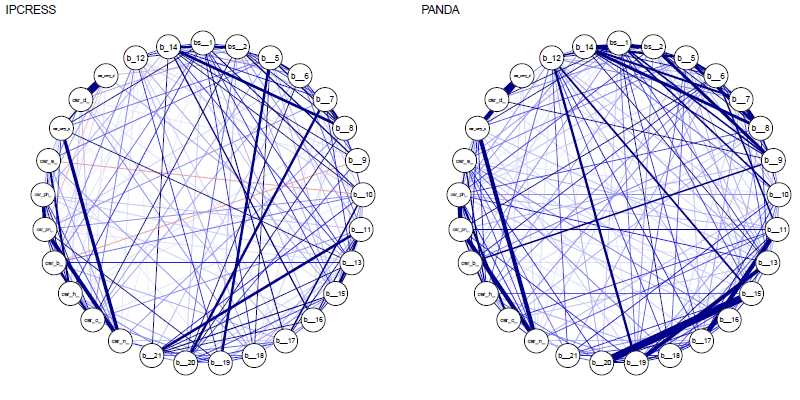


Study level networks with FGL penalty.


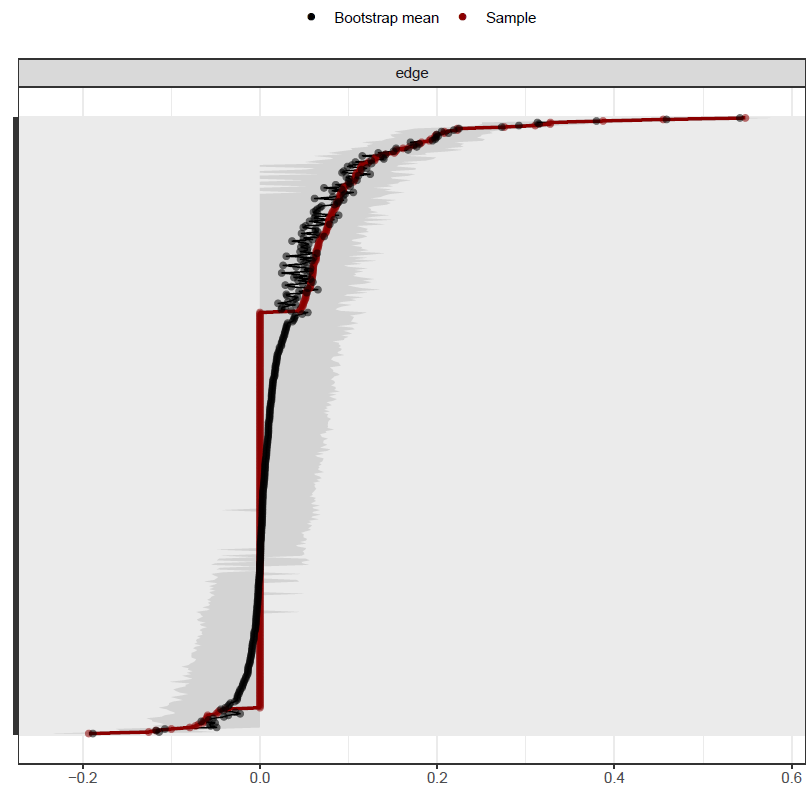


Edge weight confidence intervals.


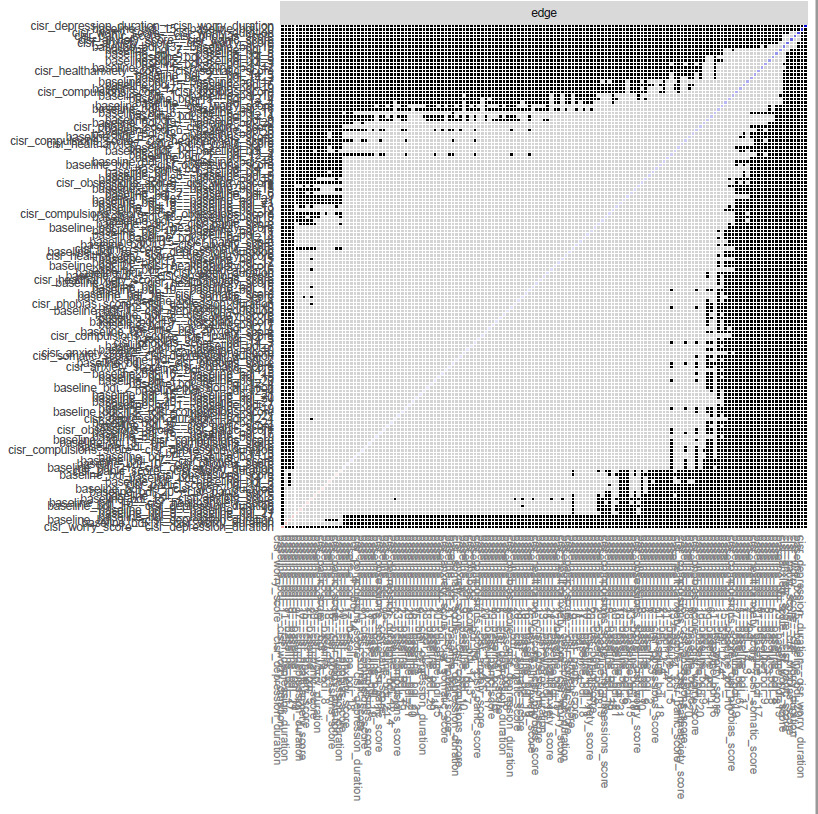


Edge weights difference test


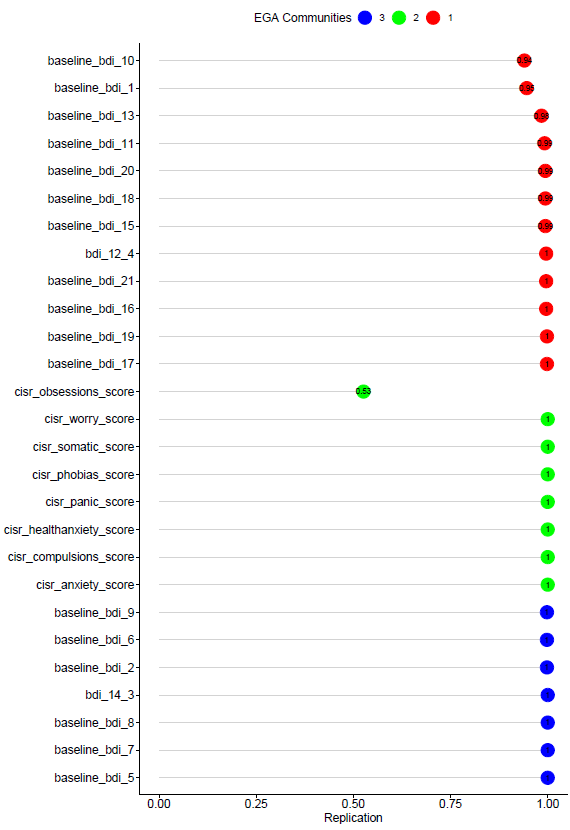


Bootstrapped communities.

|  |  | BDI-II Score | | |  |
| --- | --- | --- | --- | --- | --- |
|  |  | RMSE | R^2^ | MAE |  |
| 3 to 4 months  N=2171 | Items | 0.901 | 0.190 | 0.721 |  |
|  | Sum score | 0.899 | 0.191 | 0.718 |  |
| 6 to 8 months  N=1286 | Items | 0.894 | 0.204 | 0.719 |  |
|  | Sum score | 0.892 | 0.204 | 0.721 |  |
| 9 to 12 months  N=1102 | Items | 0.898 | 0.198 | 0.731 |  |
|  | Sum score | 0.908 | 0.176 | 0.741 |  |

Prediction modelling against BDI-II at outcome


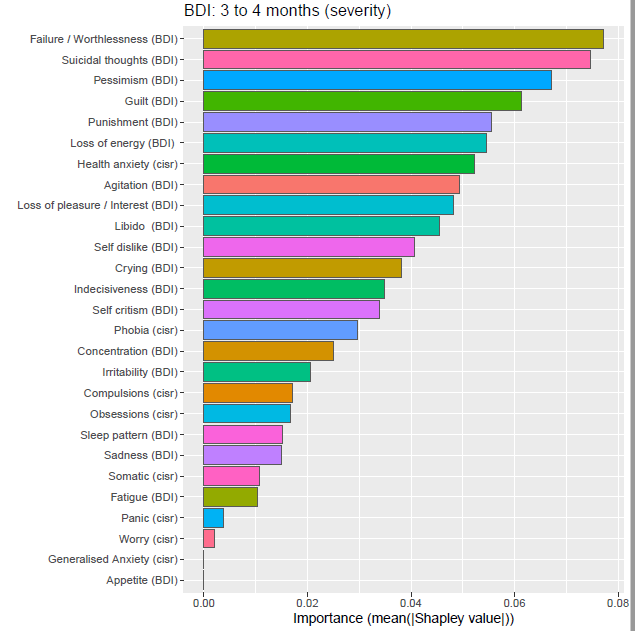

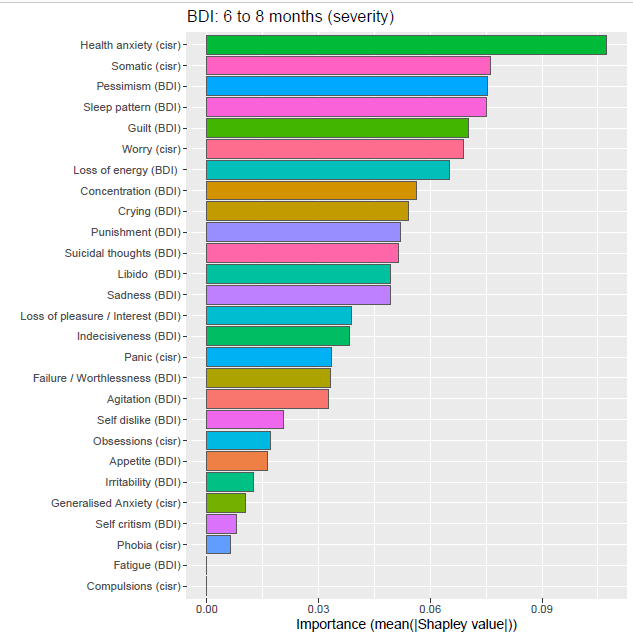

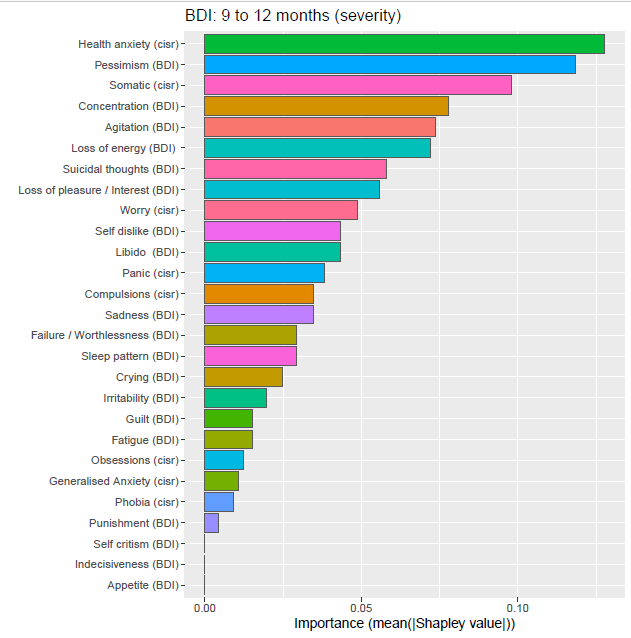


Shapley values for variable importance are plotted: (showing the difference contribution of items to predictions) for BDI-II as outcome
